# Primary Angiitis of the Central Nervous System Mimicking a Cardioembolism: Posterior Circulation Infarction With Subarachnoid Hemorrhage and Obstructive Hydrocephalus

**DOI:** 10.7759/cureus.104599

**Published:** 2026-03-03

**Authors:** Mohannad Ali, Ahmed Ameer, Samar Iltaf-Mairajuddin, Anas Hasson Alnajjar

**Affiliations:** 1 Internal Medicine, DubaiHealth, Dubai, ARE; 2 Neurology, Rashid Hospital, Dubai, ARE; 3 Radiology, DubaiHealth, Dubai, ARE

**Keywords:** cardioembolic risk factors, cns vasculitis, intracranial vasculitis, obstructive hydrocephalus, posterior circulation stroke, primary angiitis of the central nervous system (pacns), subarachanoid hemorrhage, vessel wall mri

## Abstract

Primary angiitis of the central nervous system (PACNS) is a rare inflammatory vasculopathy that frequently mimics more common cerebrovascular disorders. We report a 44-year-old woman who presented with acute posterior circulation stroke symptoms consistent with lateral medullary syndrome. Initial neuroimaging revealed a right cerebellar infarct with minimal non-aneurysmal subarachnoid hemorrhage and multifocal intracranial arterial narrowing. Concomitant atrial fibrillation and a patent foramen ovale initially suggested a cardioembolic source; however, subsequent high-resolution vessel wall magnetic resonance imaging (VW-MRI) demonstrated concentric enhancement of multiple intracranial vessels, supporting an inflammatory vasculopathy. Extensive infectious and autoimmune workups were unremarkable. Although brain biopsy remains the diagnostic gold standard, it was declined by the patient, and the diagnosis was established on clinical and radiological grounds. The clinical course was complicated by obstructive hydrocephalus requiring external ventricular drainage, and initiation of anticoagulation was deferred, given the hemorrhagic component and evolving neurological status. Treatment with high-dose corticosteroids followed by maintenance immunosuppression resulted in clinical and radiological improvement without recurrent ischemic or hemorrhagic events. This case highlights the diagnostic complexity of PACNS presenting with concurrent ischemic and hemorrhagic stroke and underscores the value of vessel wall imaging in distinguishing inflammatory vasculopathy from competing embolic etiologies.

## Introduction

Primary angiitis of the central nervous system (PACNS) is a rare, idiopathic inflammatory vasculitis confined to the brain and spinal cord, characterized histopathologically by granulomatous or lymphocytic inflammation of intracranial vessels in the absence of systemic vasculitis or infection [1,2]. The estimated annual incidence is approximately 2.4 cases per million, with peak presentation between the fourth and sixth decades of life [3]. Owing to its rarity and protean clinical manifestations, PACNS remains a formidable diagnostic challenge and is frequently underrecognized or misattributed to more common cerebrovascular conditions.

The clinical spectrum of PACNS is broad and often nonspecific, encompassing chronic headache, encephalopathy, cognitive decline, focal neurological deficits, seizures, and ischemic stroke [1,2]. Posterior circulation involvement and brainstem syndromes are less commonly reported initial presentations [3]. In addition to ischemic infarction, PACNS may manifest with intracranial hemorrhage, including subarachnoid hemorrhage, or with simultaneous ischemic and hemorrhagic events, thereby further complicating diagnostic evaluation and therapeutic decision-making [2,3].

Diagnostic criteria proposed by Calabrese and Mallek emphasize an acquired, otherwise unexplained neurologic deficit; supportive angiographic and/or histopathologic evidence of CNS arteritis; and exclusion of systemic vasculitis or secondary causes [4]. Neuroimaging is central to the diagnostic workup. Conventional angiography typically demonstrates multifocal segmental narrowing and dilatation of intracranial vessels; however, these findings lack specificity and overlap substantially with reversible cerebral vasoconstriction syndrome (RCVS) and intracranial atherosclerosis [5]. High-resolution vessel wall magnetic resonance imaging (VW-MRI) has emerged as an important adjunctive modality, enabling direct visualization of vessel wall inflammation and improving discrimination between inflammatory vasculopathies and their mimics [6,7]. Despite advances in imaging, brain and leptomeningeal biopsy remains the diagnostic gold standard; however, its invasive nature and the segmental distribution of vascular involvement may limit sensitivity and practical applicability in selected cases [8].

We report a rare and diagnostically challenging case of PACNS presenting with acute posterior circulation stroke characterized by lateral medullary features, concomitant non-aneurysmal subarachnoid hemorrhage, and subsequent obstructive hydrocephalus requiring neurosurgical intervention [9].

## Case presentation

A 44-year-old woman with a remote history of resolved Bell’s palsy presented to the emergency department with the sudden onset of dizziness, difficulty swallowing, facial asymmetry, slurred speech, and nausea. Symptoms began approximately three hours prior to presentation after she awoke from sleep in her usual state of health. She had no known chronic medical conditions and was not taking regular medications.

**Figure 1:**
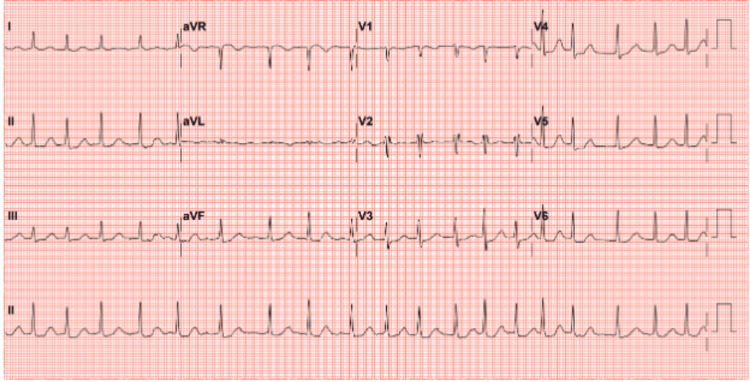
The electrocardiogram shows atrial fibrillation with rapid ventricular response.

On arrival, her blood pressure was 123/69 mmHg, pulse rate 120 beats per minute with an irregularly irregular rhythm, respiratory rate 24 breaths per minute, temperature 36.9°C, and oxygen saturation was maintained on room air. ECG showed atrial fibrillation with rapid ventricular response (Figure 1). She appeared unwell, sitting upright with copious oral secretions and mild respiratory distress. She was alert, oriented, and able to follow simple and complex commands.

Neurological examination revealed dysarthria and right-sided upper motor neuron facial weakness. Extraocular movements were intact, though horizontal nystagmus was present on right gaze. Motor and sensory examinations were normal, with preserved tone and strength in all limbs. Cerebellar testing demonstrated right-sided dysmetria on finger-to-nose and heel-to-shin testing. Gait was not assessed due to severe dizziness. Cardiovascular examination revealed an irregularly irregular pulse, while respiratory and abdominal examinations were unremarkable.

**Figure 2:**
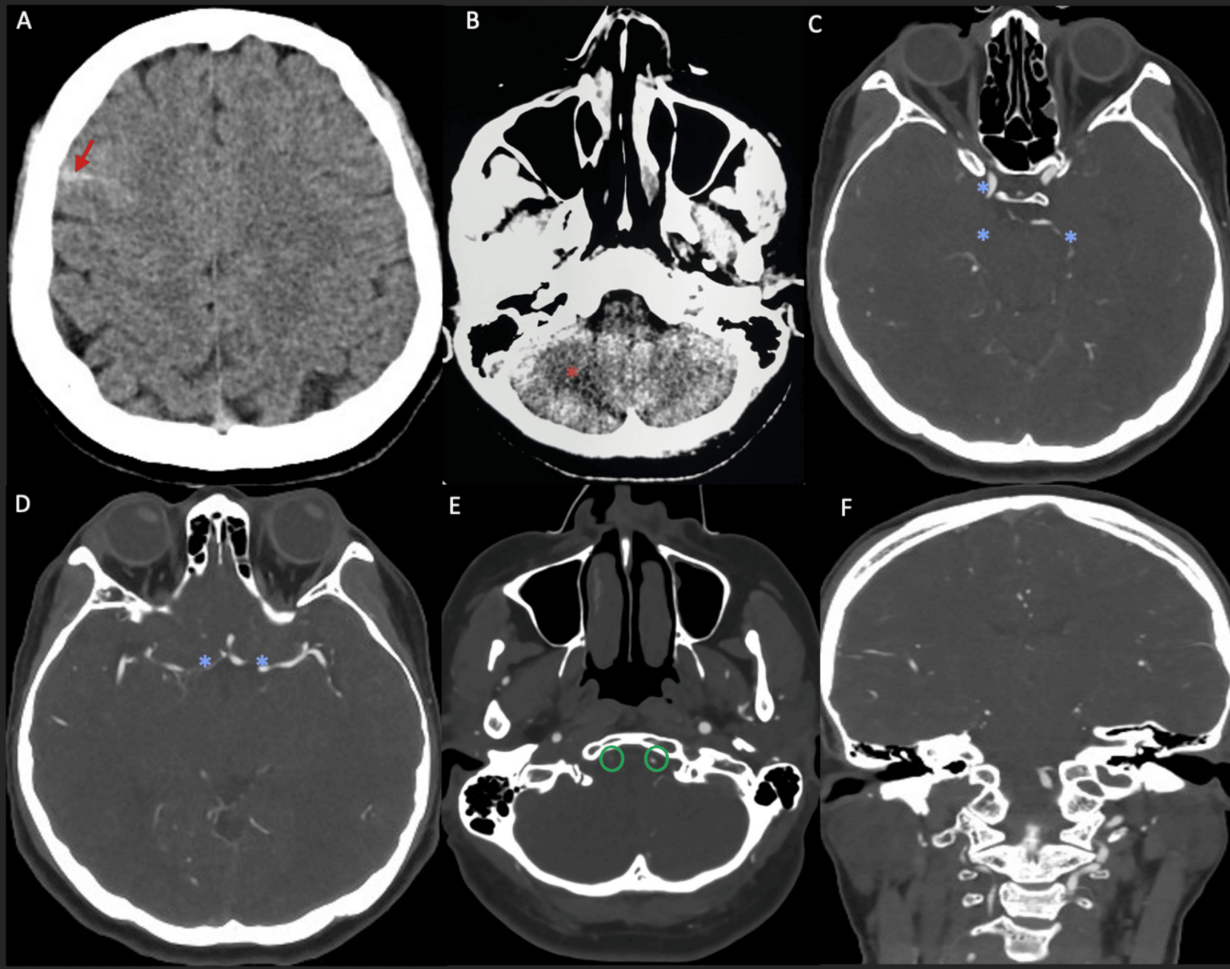
Non-contrast CT of the brain and CT angiography at admission (A–F) (A, B) Non-contrast CT of the brain demonstrates a small subarachnoid hemorrhage in the right high frontal region (red arrow) and an ill-defined hypodensity within the right cerebellar hemisphere (orange asterisk), concerning for acute infarction. (C–F) CT angiography shows multifocal segmental narrowing of the right internal carotid artery, predominantly involving the cavernous and supraclinoid segments, with additional multifocal narrowing of several vessels of the circle of Willis, including the bilateral anterior cerebral (A1), middle cerebral (M1), and posterior cerebral arteries (blue asterisks). Focal areas of narrowing are also noted in the distal segments of both vertebral arteries, with complete occlusion of the distal right vertebral artery and non-opacification of the right posterior inferior cerebellar artery (PICA) (green circles).

An urgent non-contrast CT of the brain (Figure 2) demonstrated minimal subarachnoid hemorrhage in the right high frontal region and an ill-defined hypodensity in the right cerebellar hemisphere consistent with acute infarction. CT angiography (Figure 2) showed multifocal narrowing of the right internal carotid artery at the cavernous and supraclinoid segments, with additional multifocal narrowing of the circle of Willis vessels. Intravenous thrombolysis was contraindicated due to the presence of subarachnoid hemorrhage, and neurosurgical consultation advised conservative management.

**Figure 3:**
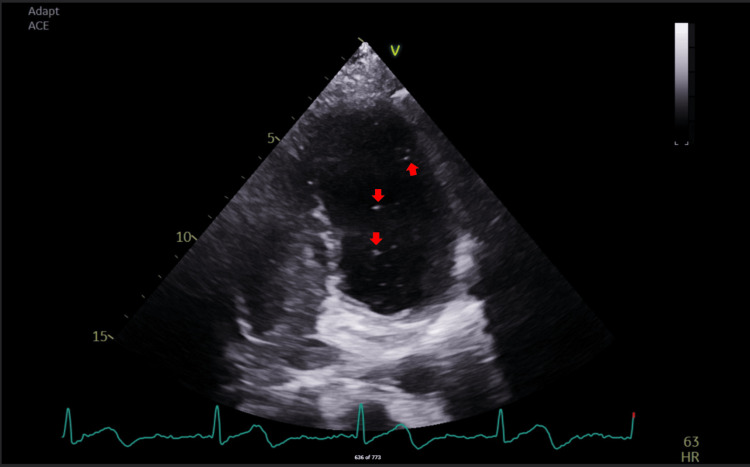
Transthoracic echocardiography (apical four-chamber view) shows microbubbles within the left ventricle following the administration of agitated saline contrast (bubble study; red arrows).

Initial laboratory investigations are summarized in Table 1. These demonstrated mild leukocytosis with neutrophil predominance, elevated troponin T, and mild hypokalemia. Cardiology was consulted regarding her atrial fibrillation with rapid ventricular response, and rate control was initiated with recommendations to start anti-therapeutic anti-coagulation once cleared by the neurology team. Transthoracic echocardiography (Figure 3) demonstrated a positive bubble study suggestive of a small patent foramen ovale. Given the above findings, the patient was admitted with an initial impression of CNS vasculitis vs. reversible cerebral vasoconstriction syndrome (RCVS); no anti-platelets or anti-coagulation was initiated on admission.

**Table 1:** Initial laboratory investigations on admission

TEST	RESULT	REFERENCE RANGE
Hemoglobin (Hb)	13.2 g/dL	12.0 – 15.0 g/dL
White blood cell count (WBC)	12.5 × 10³/µL	3.6 – 11.0 × 10³/µL
Random glucose	146 mg/dL	65 – 140 mg/dL
Troponin T	43 ng/L	<14 ng/L
N-terminal pro–B-type natriuretic peptide (NT-proBNP)	25.2 pg/mL	<125 pg/mL
International normalized ratio (INR)	1.0	0.8 – 1.1
HbA1c	4.9%	<5.7%
Thyroid-stimulating hormone (TSH)	0.446 µIU/mL	0.27 – 4.20 µIU/mL
Free thyroxine (T4)	20.6 pmol/L	12 – 22 pmol/L
Creatinine	0.70 mg/dL	0.5 – 0.90 mg/dL
Estimated glomerular filtration rate (eGFR)	110 mL/min/1.73 m²	>60 mL/min/1.73 m²
Urea	25 mg/dL	12 – 40 mg/dL
Potassium	3.2 mmol/L	3.4 – 4.5 mmol/L
Sodium	138 mmol/L	136 – 145 mmol/L
Bicarbonate (HCO₃⁻)	20.8 mmol/L	20 – 28 mmol/L
Chloride	102 mmol/L	98 – 108 mmol/L
Alanine aminotransferase (ALT)	39 U/L	0 – 31 U/L
Albumin	4.4 g/dL	3.9 – 5.0 g/dL
Alkaline phosphatase (ALP)	66 U/L	35 – 104 U/L
Total bilirubin	0.32 mg/dL	0 – 1.2 mg/dL
Low-density lipoprotein (LDL) cholesterol	85 mg/dL	<115 mg/dL
High-density lipoprotein (HDL) cholesterol	72 mg/dL	>48 mg/dL
Total cholesterol	169 mg/dL	<190 mg/dL

**Figure 4:**
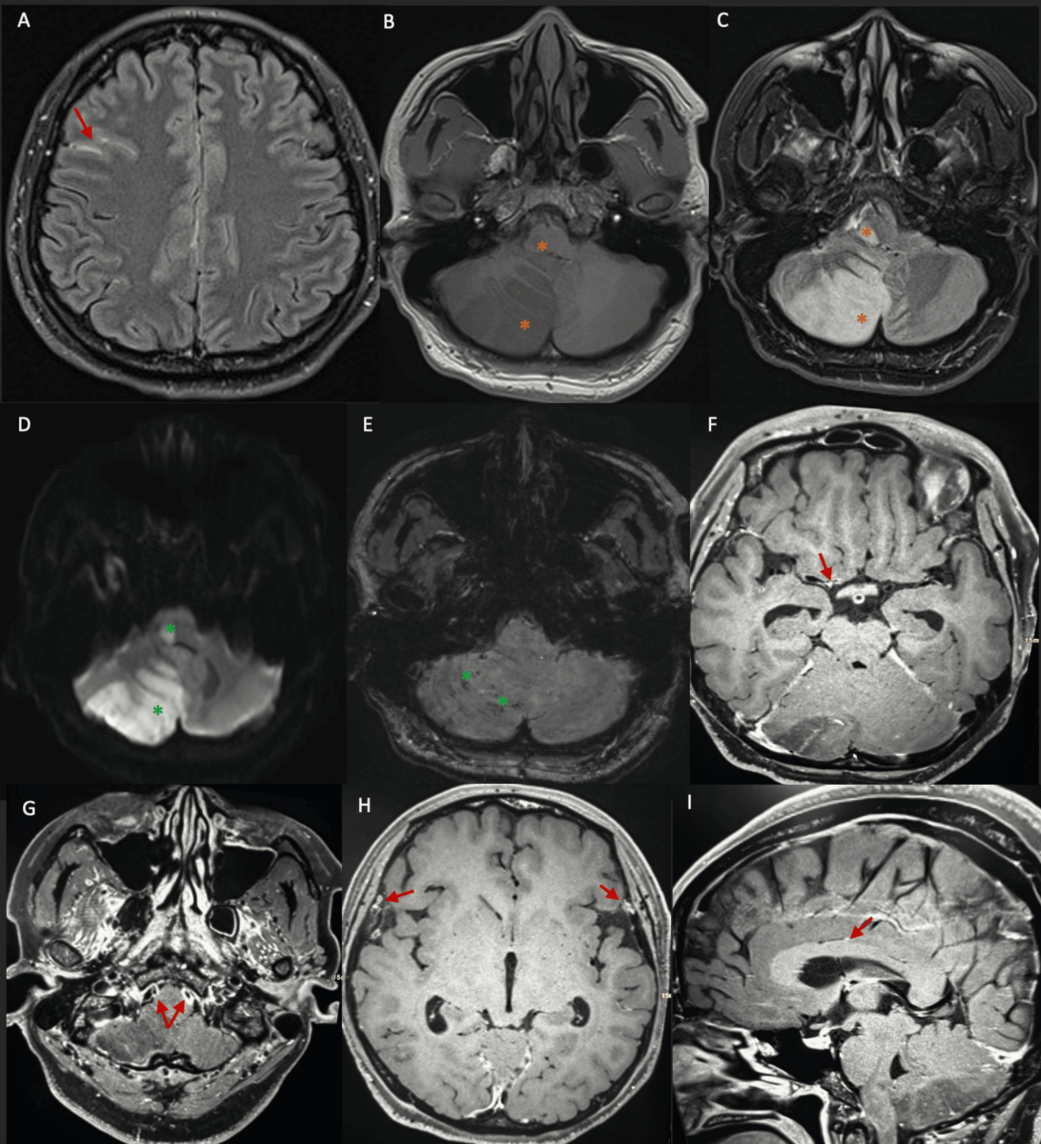
An MRI of the brain with contrast and vessel wall imaging (A-F) (A) Fluid-attenuated inversion recovery (FLAIR) with contrast images demonstrating minimal right frontal subarachnoid hemorrhage (red arrow). (B-E) Large areas of low T1 (B) and high T2/FLAIR (C) signal changes involving the right cerebellar hemisphere and right lateral medulla (orange asterisks). The corresponding areas demonstrate diffusion restriction on diffusion-weighted imaging (DWI) (D) and minimal blooming on susceptibility-weighted imaging (SWI) sequences (E) (green asterisks), suggesting acute infarcts with microhemorrhages. (F-I) High-resolution vessel wall imaging demonstrates smooth, moderate luminal narrowing with concentric wall thickening and enhancement involving multiple intracranial arteries, including the bilateral vertebral arteries, the supraclinoid segment of the right internal carotid artery, the right M1 segment, bilateral middle cerebral artery cortical branches, and the pericallosal branches of the right anterior cerebral artery (red arrows), findings consistent with inflammatory small- and medium-vessel vasculitis.

An MRI of the brain with contrast and vessel wall imaging (Figure 4) demonstrated acute infarcts involving the right cerebellar hemisphere and right lateral medulla, minimal subarachnoid hemorrhage, mild hydrocephalus, and concentric enhancement of multiple intracranial vessels, consistent with intracranial vasculitis. Extensive autoimmune, infectious, and thrombophilia workups were unremarkable (Table 2). Serologic testing for HIV, hepatitis B, hepatitis C, and syphilis was negative. In conjunction with a rheumatology consultation, a diagnosis of primary angiitis of the central nervous system was established, and intravenous methylprednisolone 1000 mg once daily for five days was initiated.

**Table 2:** Autoimmune and thrombophilia workup *ENA profile includes antibodies associated with Sjögren syndrome, dermatomyositis, systemic and limited systemic sclerosis, and drug-induced systemic lupus erythematosus.

TEST	RESULT	REFERENCE RANGE
Anti-β2 glycoprotein I IgG	18.5 CU	Negative <20 CU; Positive ≥20 CU
Anti-β2 glycoprotein I IgM	1.5 CU	Negative <20 CU; Positive ≥20 CU
Anti-cardiolipin IgG	8.8 CU	Negative <20 CU; Positive ≥20 CU
Anti-cardiolipin IgM	5.5 CU	Negative <20 CU; Positive ≥20 CU
Anti–double-stranded DNA antibodies	<10 IU/mL	Negative <100 IU/mL; Positive ≥100 IU/mL
Proteinase 3 antibody (PR3)	<2.3 CU	Negative <20 CU; Positive ≥20 CU
Myeloperoxidase antibody (MPO)	<3.2 CU	Negative <20 CU; Positive ≥20 CU
Anti-nuclear antibody (ANA)	Negative (<1:100)	Negative <1:100; Positive ≥1:100
Erythrocyte sedimentation rate (ESR)	14 mm/hr	2 – 37 mm/hr
Lupus anticoagulant	Negative	Negative
Rheumatoid factor	<10 IU/mL	<14 IU/mL
Factor II (prothrombin)	Normal	Normal
Factor V (Leiden mutation)	Normal	Normal
Protein C activity	127%	70% – 140%
Protein S activity	101%	60% – 160%
Antithrombin III activity	113%	80% – 130%
Activated protein C resistance	206 sec	120 – 300 sec
Phosphatidylserine IgG antibodies	4.20	<10.0
Phosphatidylserine IgM antibodies	2.80	<10.0
Extractable nuclear antigen (ENA) profile*	<0.1 (Ratio)	Negative <1.0; Borderline 1.0–2.0; Positive 2.0–5.0; High positive ≥5.0

**Figure 5:**
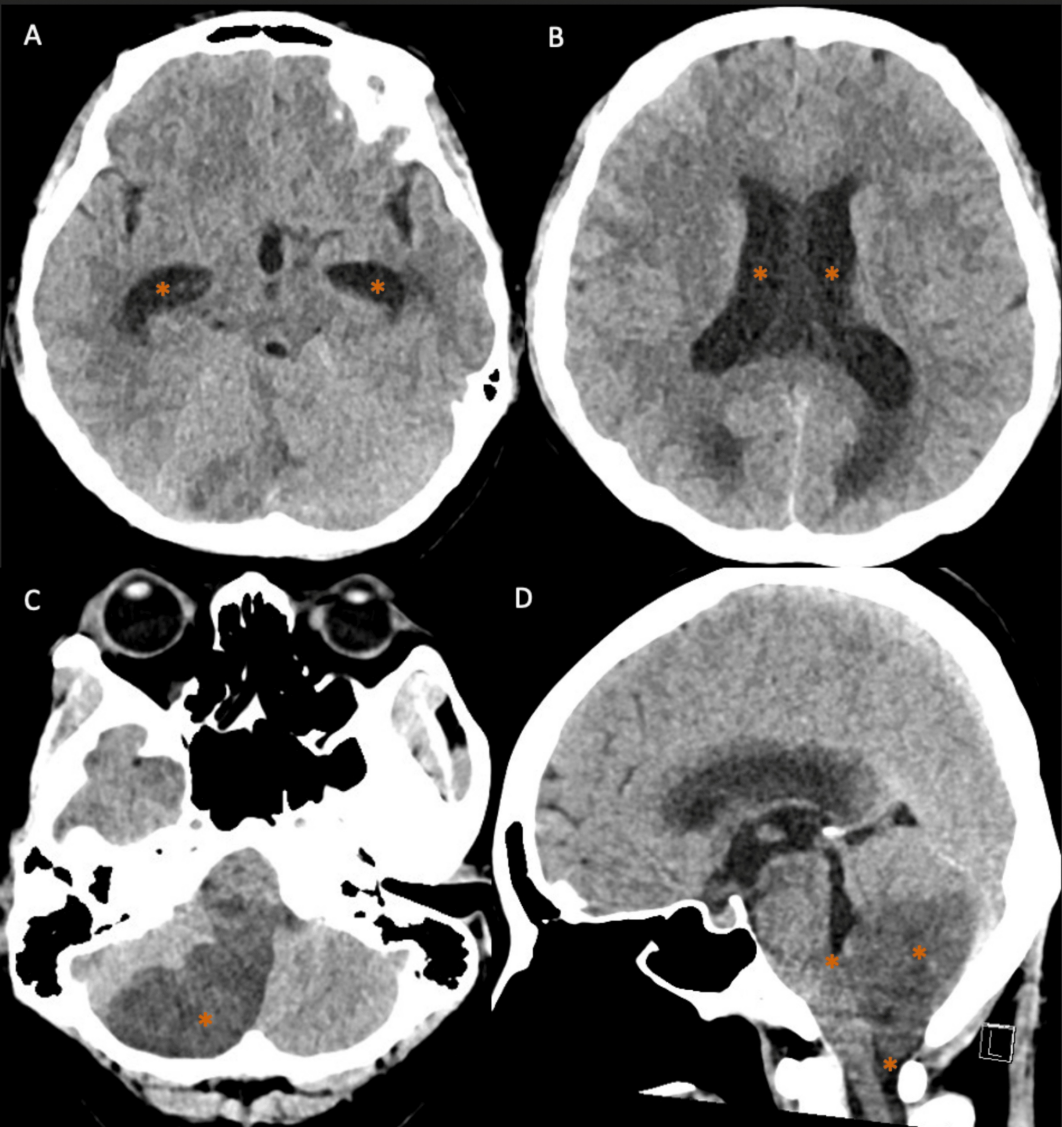
Follow-up CT of the brain following neurological deterioration (A-D) Well-established right cerebellar infarct in the posterior inferior cerebellar artery (PICA) territory, with associated edema and evolving obstructive hydrocephalus secondary to compression of the fourth ventricle and brainstem, mild crowding at the foramen magnum, and tonsillar descent measuring approximately 5 mm (orange asterisks).

The patient later developed worsening neurological symptoms, including hiccups, increased drooling, right-sided ptosis, miosis, and worsening nystagmus. Repeat CT of the brain (Figure 5) demonstrated evolving obstructive hydrocephalus with compression of the fourth ventricle and brainstem. An external ventricular drain was urgently inserted, and the patient was managed in the intensive care unit. Cerebrospinal fluid (CSF) obtained via the external ventricular drain on the same day (one day after initiation of intravenous methylprednisolone) showed protein 14 mg/dL, glucose 93 mg/dL, white blood cells <5/µL, and red blood cells 200/µL. Gram stain and culture were negative, and a CSF meningitis/encephalitis polymerase chain reaction (PCR) panel was negative.

**Figure 6:**
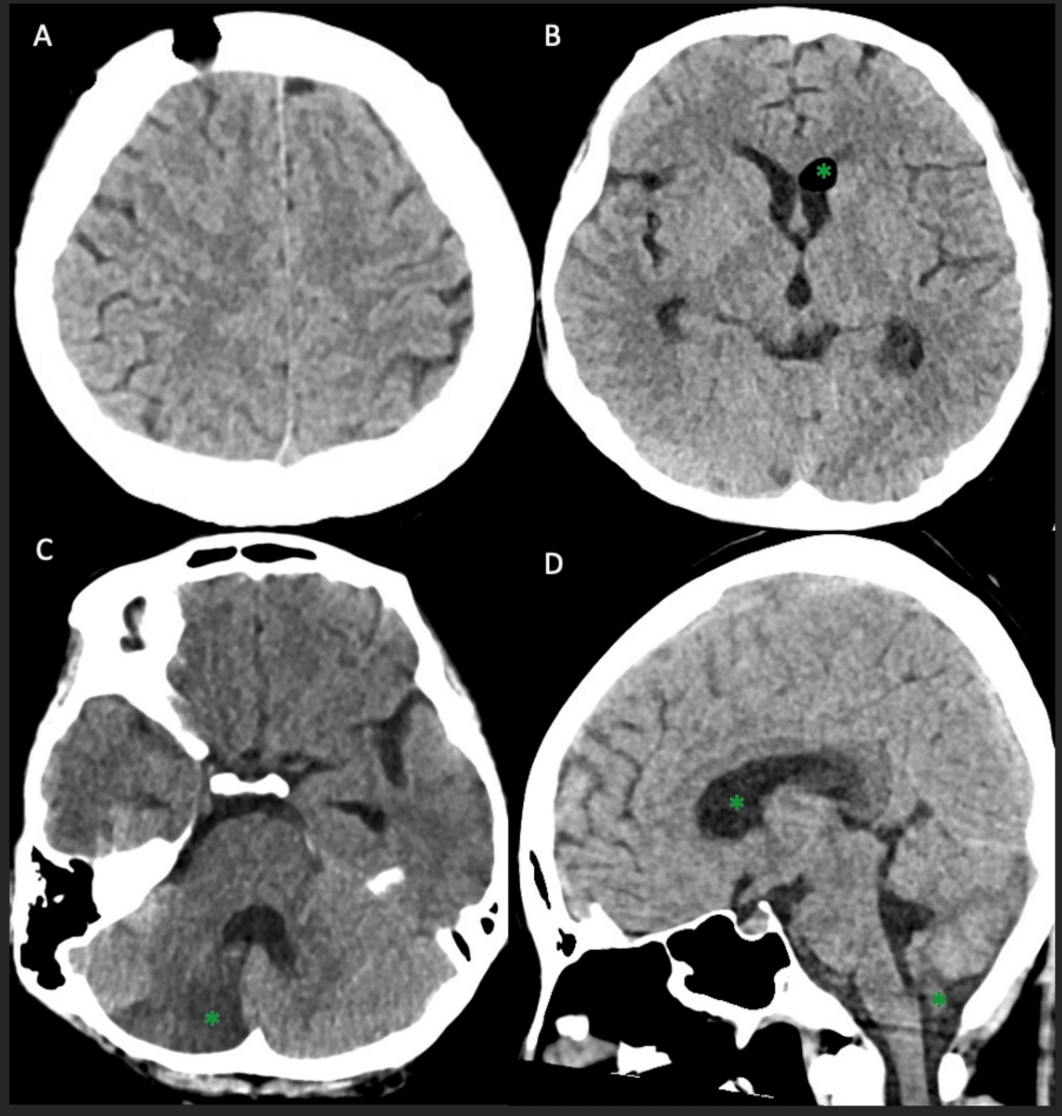
Follow-up CT of the brain prior to discharge (A-D) Established right cerebellar infarct in the posterior inferior cerebellar artery (PICA) territory, with interval resolution of the previously noted right frontal subarachnoid hemorrhage, obstructive hydrocephalus, posterior fossa edema, and tonsillar descent. Interval development of a small air pocket within the frontal horn of the left lateral ventricle is noted, consistent with post-procedural change (green asterisks).

Following completion of a five-day course of intravenous methylprednisolone, she was transitioned to oral prednisone 50mg once daily. The external ventricular drain was removed after imaging confirmed resolution of hydrocephalus (Figure 6). Therapeutic anticoagulation was initiated after neurosurgical clearance. The patient was discharged on prednisolone 50mg once daily and apixaban 2.5mg twice daily, with ongoing outpatient follow-up arranged.

At outpatient follow-up, the patient remained significantly disabled (modified Rankin Scale (mRS) score 4). She required a wheelchair and continued to exhibit right-sided hemiparesis with ataxia, nasal dysarthria, impaired smooth pursuit, left abducens palsy with diplopia, and persistent dysphagia requiring nasogastric feeding. She was receiving prednisolone 50 mg daily and apixaban 2.5 mg twice daily. Azathioprine 50 mg daily was initiated as maintenance immunosuppression, prednisolone was tapered to 40 mg daily, and apixaban was increased to 5 mg twice daily.

**Figure 7:**
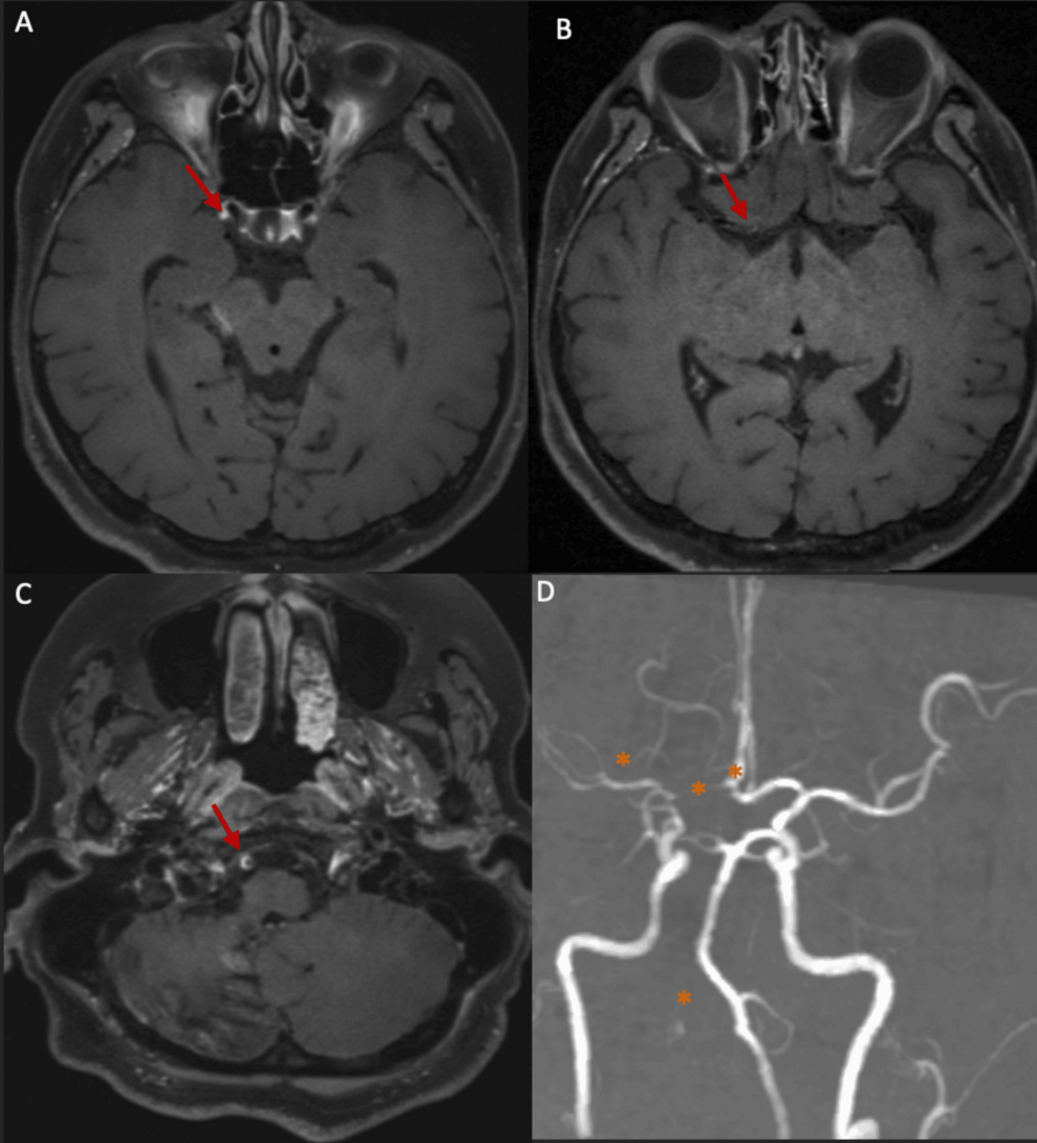
Follow-up contrast-enhanced brain MRI with high-resolution vessel wall imaging ((A–D) (A–B) Post-contrast T1-weighted black-blood high-resolution vessel wall images demonstrate interval reduction in concentric wall thickening and enhancement compared with the prior study (red arrows). (C–D) Persistent luminal narrowing with residual enhancement is noted in the right vertebral artery (V4 segment) and the right middle cerebral artery (M1 segment), without new vascular territories involved (orange asterisks).

At subsequent review, she reported modest improvement in limb weakness and ataxia, although functional dependence persisted (mRS score of 4). Repeat high-resolution VW-MRI (Figure 7) demonstrated interval reduction in concentric vessel wall enhancement compared with prior imaging, consistent with radiological improvement. Persistent narrowing of the vertebral V4 segments and right supraclinoid middle cerebral artery involvement remained stable, with no new vascular territories affected. Prednisolone tapering was continued, and azathioprine was maintained. No recurrent ischemic or hemorrhagic events were observed during follow-up. Table 3 outlines the detailed timeline of the patient’s hospital course, summarizing key clinical events, diagnostic findings, and therapeutic interventions.

**Table 3:** Timeline of the patient's clinical course

DATE	CLINICAL EVENT
06/12/2025	Symptom onset and emergency department admission
06/12/2025	CT of the brain and CT angiography
09/12/2025	MRI of the brain with vessel wall imaging
09/12/2025	Initiation of intravenous methylprednisolone
10/12/2025	Neurological deterioration
10/12/2025	CT of the brain showing obstructive hydrocephalus
10/12/2025	External ventricular drain insertion
25/12/2025	External ventricular drain removal
29/12/2025	Initiation of therapeutic anticoagulation
02/01/2026	Discharge from the hospital
27/01/2026	First outpatient follow-up. Tapering of the steroid, nonsteroidal immunosuppressive agent added, and anti-coagulation escalated to full dose
10/02/2026	Second outpatient follow-up. Further tapering of the steroid

## Discussion

PACNS is a rare single-organ vasculitis that lacks pathognomonic clinical features and is frequently misdiagnosed as a more common cerebrovascular disease [1,2]. Diagnostic criteria proposed by Calabrese and Mallek emphasize (1) an acquired, otherwise unexplained neurologic deficit, (2) evidence of an arteritic process on angiography and/or histopathology, and (3) exclusion of systemic vasculitis or secondary causes such as infection or malignancy [4]. In practice, meeting these criteria requires a structured approach because inflammatory markers and autoimmune serologies may be normal, and clinical presentations overlap with non-inflammatory vasculopathies [1,2].

In our patient, the initial syndrome was consistent with posterior circulation ischemic stroke with lateral medullary features, and the concurrent atrial fibrillation and patent foramen ovale offered a plausible competing cardioembolic mechanism. However, several features supported an inflammatory vasculopathy: multifocal segmental intracranial narrowing on CTA with distal vertebral involvement, infarction affecting more than one posterior circulation structure with microhemorrhagic elements, and the presence of non-aneurysmal subarachnoid hemorrhage. These findings prompted evaluation for PACNS and its mimics, particularly RCVS, intracranial atherosclerotic disease, arterial dissection, and infectious vasculopathies [5].

High-resolution HR-VWI is particularly useful because it can directly characterize the vessel wall rather than relying solely on luminal caliber. In PACNS, HR-VWI commonly shows smooth, concentric wall thickening with homogeneous enhancement, reflecting inflammatory infiltration. In contrast, intracranial atherosclerosis more often produces eccentric plaque enhancement, while RCVS tends to show absent or less prominent wall enhancement despite multifocal luminal narrowing; importantly, serial HR-VWI can demonstrate interval improvement with resolution of vasoconstriction or treatment response [6,7]. In our case, the vessel wall enhancement pattern and interval reduction on follow-up HR-VWI after immunosuppressive therapy supported an inflammatory vasculitis process and increased confidence in the working diagnosis of PACNS [6,7].

CSF analysis is abnormal in most patients with PACNS, typically demonstrating mild lymphocytic pleocytosis and/or elevated protein [8]. Nevertheless, lumbar puncture may be unsafe when raised intracranial pressure or posterior fossa mass effect is present, which is relevant in cases complicated by hydrocephalus. In our patient, CSF obtained via an An external ventricular drain after steroid initiation was largely unremarkable (white blood cells <5/µL) with negative Gram stain/culture and a negative meningitis/encephalitis PCR panel. Although brain and leptomeningeal biopsy remains the diagnostic gold standard, its sensitivity is limited by segmental (“skip”) involvement, and biopsy is not always feasible or acceptable to patients [8]. Accordingly, modern diagnostic pathways increasingly integrate advanced neuroimaging alongside clinical and laboratory exclusion of secondary causes [8].

The hemorrhagic component is a key aspect of this case. Hemorrhagic manifestations, including subarachnoid hemorrhage and intraparenchymal hemorrhage, are described in PACNS and may reflect inflammatory injury and fragility of the vessel wall or hemorrhagic transformation within infarcted tissue [1-3]. From a diagnostic standpoint, hemorrhage also heightens concern for RCVS because non-aneurysmal subarachnoid haemorrhage can occur in RCVS cohorts, reinforcing the importance of careful clinical and imaging correlation prior to committing to immunosuppressive therapy [5,6].

Our patient additionally developed obstructive hydrocephalus requiring external ventricular drainage, a rare but clinically significant complication. Hydrocephalus in primary CNS vasculitis has been reported and may arise from impaired CSF dynamics related to inflammation and/or obstruction of CSF pathways [9]. This case reinforces the need for close neurological monitoring and repeat neuroimaging in posterior circulation PACNS, particularly when symptoms progress.

During the period of neurological deterioration, the patient developed a left abducens palsy that was not anatomically attributable to the right posterior inferior cerebellar artery territory infarction. No additional pontine ischemia was identified on repeat imaging. We believe the most plausible mechanism was a false localizing cranial nerve VI palsy secondary to obstructive hydrocephalus and posterior fossa mass effect. The documented fourth ventricular compression, brainstem crowding, and tonsillar descent likely resulted in raised intracranial pressure and stretch injury to the abducens nerve along its long intracranial course. Given the known vulnerability of cranial nerve VI to traction in the setting of increased intracranial pressure, the persistent deficit despite radiographic resolution of hydrocephalus may reflect residual axonal injury sustained during the acute phase.

Finally, concurrent atrial fibrillation and subarachnoid hemorrhage created a therapeutic dilemma regarding anticoagulation. The hemorrhagic component precluded thrombolysis and required deferral of therapeutic anticoagulation initially. Anticoagulation was introduced after radiographic stability and neurosurgical clearance, with escalation once bleeding risk was felt to have sufficiently diminished. This emphasizes the importance of multidisciplinary decision-making (neurology, rheumatology, neurosurgery, and cardiology) in complex mixed ischemic-hemorrhagic presentations.

## Conclusions

This case illustrates a rare presentation of PACNS manifesting as a posterior circulation ischemic stroke, subarachnoid hemorrhage, and obstructive hydrocephalus. It highlights the growing diagnostic utility of HR-VWI, particularly when a brain biopsy is not feasible or is declined. Early multidisciplinary management, incorporating immunosuppression and timely neurosurgical intervention, may be crucial to achieving favorable outcomes. Prospective multicenter studies are needed to better define the comparative role of advanced vessel wall imaging versus biopsy and to establish standardized recommendations for the selection and duration of immunosuppressive therapy.
